# Observational cohort study of rilpivirine (RPV) utilization in Europe

**DOI:** 10.1186/s12981-022-00457-0

**Published:** 2022-08-06

**Authors:** Alessandro Cozzi-Lepri, Lars Peters, Annegret Pelchen-Matthews, Bastian Neesgaard, Stephane De Wit, Isik Somuncu Johansen, Simon Edwards, Christoph Stephan, Georgios Adamis, Therese Staub, Alexandra Zagalo, Pere Domingo, Daniel Elbirt, Katharina Kusejko, Johanna Brännström, Dzmitry Paduta, Tatyana Trofimova, Janos Szlavik, Kai Zilmer, Marcello Losso, Veerle Van Eygen, Helen Pai, Jens Lundgren, Amanda Mocroft, for the EuroSIDA Study Group, A. Harxhi, M. Losso, M. Kundro, B. Schmied, I. Karpov, A. Vassilenko, D. Paduto, V. M. Mitsura, N. Clumeck, S. De Wit, M. Delforge, V. Hadziosmanovic, J. Begovac, L. Machala, D. Jilich, J. Gerstoft, C. Pedersen, D. Sedlacek, G. Kronborg, T. Benfield, I. S. Johansen, L. Ostergaard, L. Wiese, N. F. Moller, L. N. Nielsen, K. Zilmer, Jelena Smidt, I. Aho, J. P. Viard, P. M. Girard, C. Pradier, E. Fontas, C. Duvivier, J. Rockstroh, O. Degen, C. Hoffmann, H. J. Stellbrink, C. Stefan, J. Bogner, G. Fätkenheuer, N. Chkhartishvili, H. Sambatakou, G. Adamis, N. Paissios, V. Uzdaviniene, T. Staub, S. Dragas, P. Reiss, J. Trajanovska, D. H. Reikvam, A. Maeland, J. Bruun, B. Knysz, B. Szetela, M. Inglot, E. Bakowska, R. Flisiak, A. Grzeszczuk, M. Parczewski, K. Maciejewska, B. Aksak-Was, M. Beniowski, E. Mularska, E. Jablonowska, J. Kamerys, K. Wojcik, I. Mozer-Lisewska, B. Rozplochowski, A. Zagalo, R. Radoi, C. Oprea, A. Yakovlev, T. Trofimora, I. Khromova, E. Kuzovatova, E. Borodulina, E. Vdoushkina, J. Ranin, J. Tomazic, J. M. Miro, M. Laguno, E. Martinez, F. Garcia, J. L. Blanco, M. Martinez-Rebollar, J. Mallolas, P. Callau, J. Rojas, S. Moreno, S. del Campo, A. Jou, R. Paredes, J. Puig, J. M. Llibre, J. R. Santos, P. Domingo, M. Gutierrez, G. M. Mateo, A. Sambeat, J. M. Laporte, V. Svedhem, A. Thalme, A. Sonnerborg, L. Flamholc, K. Kusejko, R. Weber, A. Calmy, H. Furrer, M. Battegay, P. Schmid, A. Kuznetsova, J. Mikhalik, M. Sluzhynska, A. Milinkovic, A. M. Johnson, E. Simons, S. Edwards, A. M. Phillips, A. Johnson, A. Mocroft, C. Orkin, A. Winston, A. Clarke, C. Leen

**Affiliations:** 1 Centre for Clinical Research, Epidemiology, Modelling and Evaluation (CREME), Institute for Global Health, University College London, Rowland Hill St, London, NW3 2PF UK; 2 CHIP, Rigshospitalet, University of Copenhagen, Copenhagen, Denmark; 3 Department of Infectious Diseases, CHU Saint-Pierre, Université Libre de Bruxelles, Brussels, Belgium; 4 Department of Infectious Diseases, Odense University Hospital, Odense, Denmark; 5 Mortimer Market Centre, Department of HIV, London, UK; 6 Infectious Diseases Unit, Goethe-University Hospital, Frankfurt, Germany; 7 1St Department of Internal Medicine and Infectious Diseases Unit, General Hospital of Athens G. Gennimatas, Athens, Greece; 8 Centre Hospitalier de Luxembourg, Service des Maladies Infectieuses, Luxembourg City, Luxembourg; 9 Department of Infectious Diseases, Santa Maria University Hospital, Lisbon, Portugal; 10 Hospital de La Santa Creu I Sant Pau, Barcelona, Spain; 11 Allergy, Immunology and HIV Unit | Kaplan, Medical Center, Rehovot, Israel; 12 Division of Infectious Diseases, University Hospital Zürich, Zurich, Switzerland; 13 Department of Infectious Diseases, Venhälsan Södersjukhuset, Stockholm, Sweden; 14 Gomel Regional Centre for Hygiene, Gomel, Belarus; 15 Novgorod Centre for AIDS Prevention and Control, Novgorod the Great, Russia; 16 South-Pest Hospital Centre–National Institute for Infectiology and Haematology, Budapest, Hungary; 17 West-Tallinn Central Hospital, Infectious Diseases Clinic, Talinn, Estonia; 18 Hospital J.M. Ramos Mejia, Buenos Aires, Argentina; 19 Janssen Research & Development, Beerse, Belgium; 20 Janssen Research & Development, Raritan, NJ USA

**Keywords:** Cohort Study, Europe, Edurant™, Eviplera™, Efavirenz, Real-world effectiveness

## Abstract

**Introduction:**

Data on safety and effectiveness of RPV from the real-world setting as well as comparisons with other NNRTIs such as efavirenz (EFV) remain scarce.

**Methods:**

Participants of EuroSIDA were included if they had started a RPV- or an EFV-containing regimen over November 2011-December 2017. Statistical testing was conducted using non-parametric Mann–Whitney U test and Chi-square test. A logistic regression model was used to compare participants’ characteristics by treatment group. Kaplan–Meier analysis was used to estimate the cumulative risk of virological failure (VF, two consecutive values > 50 copies/mL).

**Results:**

1,355 PLWH who started a RPV-based regimen (11% ART-naïve), as well as 333 initiating an EFV-containing regimen were included. Participants who started RPV differed from those starting EFV for demographics (age, geographical region) and immune-virological profiles (CD4 count, HIV RNA). The cumulative risk of VF for the RPV-based group was 4.5% (95% CI 3.3–5.7%) by 2 years from starting treatment (71 total VF events). Five out of 15 (33%) with resistance data available in the RPV group showed resistance-associated mutations vs. 3/13 (23%) among those in the EFV group. Discontinuations due to intolerance/toxicity were reported for 73 (15%) of RPV- vs. 45 (30%) of EFV-treated participants (p = 0.0001). The main difference was for toxicity of central nervous system (CNS, 3% vs. 22%, p < 0.001).

**Conclusion:**

Our estimates of VF > 50 copies/mL and resistance in participants treated with RPV were similar to those reported by other studies. RPV safety profile was favourable with less frequent discontinuation due to toxicity than EFV (especially for CNS).

**Supplementary Information:**

The online version contains supplementary material available at 10.1186/s12981-022-00457-0.

## Introduction

Rilpivirine (RPV) is a non-nucleoside reverse transcriptase inhibitor (NNRTI) which was approved as Edurant™ by the European Commission on 28 November 2011 for the treatment of human immunodeficiency virus type 1 (HIV-1) infection in antiretroviral treatment (ART)-naïve adult persons living with HIV (PLWH) with a baseline viral load (VL) ≤ 100,000 HIV-RNA copies/ml. RPV has been made available over the years as the active ingredient in several marketed fixed dose combination (FDC) tablets [1]. Although RPV in the FDC tablet formulation including emtricitabine (FTC) and tenofovir disoproxil fumarate (TDF) (Eviplera™) was initially approved for ART-naïve adult PLWH with a baseline VL ≤ 100,000 copies/ml only, the indication was extended in October 2013 for ART-experienced adults who are virologically suppressed with no history of virological failure (VF).

Several randomized studies [2–9] have compared the efficacy of RPV-based regimens with that of EFV-based regimens in both the ART-naïve and ART-experienced populations. Overall, these studies have shown relatively low rates of VF at 1 year (never above 15%) with no evidence for a difference in rate of VF. However, long-term virological outcomes of these regimens have been rarely examined in real-world clinical practice across Europe [10–12].

The aim of this analysis was to describe the use of RPV-containing regimens in routine clinical practice among adult PLWH followed in the EuroSIDA study. Specifically, key objectives were: (i) to evaluate factors associated with the probability of initiation of RPV- instead of EFV-containing regimens and (ii) to describe the safety and effectiveness of RPV, including evaluation of virological response and treatment-emergent resistance-associated mutations (RAMs).

## Methods

### Study design

The EuroSIDA study cohort is a prospective, observational cohort of HIV-1 infected participants from 117 clinical centers across Europe, Israel and Argentina [13]. Details regarding study design and data collection are described elsewhere [https://chip.dk/Research/Studies/EuroSIDA]. Information about adverse events are collected in the EuroSIDA cohort according to the Data Collection on Adverse events of Anti-HIV Drugs (D:A:D) definitions starting from 1998 [14], and detailed information about the cause of death is collected using the Coding of Death (CoDe) algorithm [15].

Up to March 2017, source documents from the laboratories performing resistance testing were sent to the EuroSIDA coordination center and recorded RAMs were keyed into a central database. The keyed results were subsequently double checked for correctness by a second person. After March 2017 resistance test results were no longer reported to EuroSIDA because VF was infrequent and resistance testing was very rarely perfomed.

### Data quality assurance

To ensure verification of collected data, an extensive quality assurance programme has been in place since EuroSIDA was initiated. Monitoring previously included on-site visits to all participating centers. However, as the study has evolved, new processes have been formulated including centralized monitoring, building on the risk-based monitoring used in clinical trials. In autumn 2014, EuroSIDA also transitioned from collecting data on paper forms to using the electronic case report system Research Electronic Data Capture [16]. More details together with the full ist of collect variables are provided here [13, 17].

### Study population

Participants were included if they were at least 16 years of age and they had started a RPV-containing or EFV-containing regimen over the period November 2011 to end of December 2017. November 2011 is the earliest of the dates of market availability of RPV-containing products (Edurant™, the single agent tablet, or Eviplera™, a single tablet regimen containing RPV/TDF/FTC) within the 28 countries where RPV-containing regimens were approved and marketed for use in participants with baseline VL ≤ 100,000 copies/ml (see full list of these countries in Additional file 1).

In October 2013, Eviplera™ was approved and marketed for use in ART-experienced adults who are virologically suppressed with no history of VF, and therefore people starting Eviplera™ from ART-naive or switching to Eviplera™ were also included. The last follow-up date recorded for a person in this analysis is December 31, 2017. Although FTC/RPV/tenofovir alafenamide (brand name Odefsey™) was approved in the European Union in June 2016, there was no use of this formulation in participants of this analysis. Thus, RPV mentioned throughout this work refers to RPV-containing regimens, either the single agent Edurant™ or the FDC (FTC/RPV/TDF), Eviplera™, while EFV refers to EFV-containing regimens, either the single agent (SUSTIVA/STOCRIN™) or the FDC ATRIPLA™.

All PLWH who started EFV-based regimens regardless of the level of VL were included, as a comparator group, to provide a contemporary context of utilization and outcomes (i.e. rates of VF and resistance patterns). Unfortunately, the use of EFV has waned in the clinics over time and the a priori target for the minimum number of VF events in this group required for a formal comparison was never reached over the duration of the study.

### Statistical analysis

Continuous variables were summarised using medians, minimums, maximums, and interquartile ranges as appropriate. Characteristics of participants who initiated RPV- or EFV-based regimens were described. Statistical testing was conducted to compare population medians (non-parametric Mann–Whitney U test) and chi-square test for proportions by treatment group. A logistic regression model was used to identify factors independently associated with the probability of starting RPV- vs. EFV- based regimens. Age, gender, weight, or body mass index (BMI), ethnicity, CD4 cell count, pre-treatment VL, mode of HIV transmission, co-infection with hepatitis B/C, HIV treatment status (naïve/experienced) were all identified as potential determinants of treatment initiation either with RPV or EFV. See Additional file 1: Table AF1  for a detailed codebook of variables included. Unadjusted and adjusted odds ratios (ORs) of starting RPV vs. EFV were tabulated with 95% confidence intervals (CI). Characteristics showing an association with the outcome of starting RPV with a p-value < 0.1 were considered for inclusion in the multivariable model; for categorical variables the global (type III) p-value was used to detect overall associations.

The main reason for discontinuing RPV and EFV, as reported by the treating physicians from a pre-specified list of options, were summarized and frequency compared by treatment group. Average levels of selected laboratory parameters (most recent value before stopping RPV/ EFV) were also calculated and compared.

Time to confirmed VF > 50 copies/mL in participants who started RPV-based regimens and had at least two VL measurements after starting therapy was estimated. The date of VF was defined at the time of the first of two consecutive values > 50 copies/mL after >6 months from starting the RPV- or EFV-based regimen. Due to the small number of VF events, especially in the EFV-treated group, VF and resistance accumulation analyses are only descriptive. Kaplan–Meier analysis was used to estimate the cumulative probability of VF > 50 copies/mL by 1 and 2 years with 95% CI in the RPV group alone.

There were missing data for some of the variables collected. For categorical factors, under the assumption of data missing at random, a separate group for missing values was allowed so that no participants were excluded from the analysis (the missing- indicator method). If a variable was analysed as continuous and there were missing values for this variable, participants with the missing value were instead excluded. All data were analysed using SAS version 9.4 (Cary, NC, USA).

## Results

### Study population

**Fig. 1:**
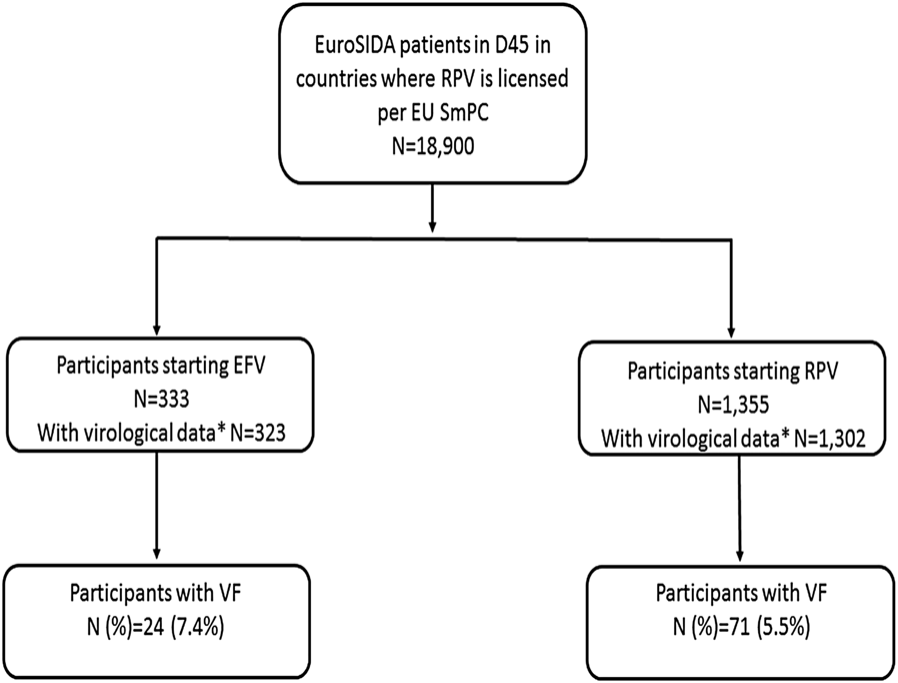
Overview of EuroSIDA participants included in the analysis. VF: first two consecutive VL > 50 copies/mL after >6 months from initiation of RPV/RFV-based regimens. *≥2 Viral load measures after baseline

Overall, as of December 31st, 2017, the number of participants within the labelled indication in EU for RPV (i.e. participants with a baseline VL of ≤ 100,000 copies/ml) newly initiating a RPV-containing regimen was 1,355, and the number of participants initiating an EFV-containing regimen was 333. A total of 144 (11%) of the RPV-based treated group were ART-naïve. Additional information regarding participants included in the RPV treatment group alone are shown in Additional file 1: Table AF2. Of the 333 included participants who started an EFV-containing regimen, 178 (55%) were ART-naïve (Fig. 1).

Table 1 shows the main characteristics of participants who started RPV or EFV at time of treatment initiation. Participants treated with RPV were older (median ages 48 vs. 39 years; p < 0.0001), had higher CD4 cell counts (median 566 vs. 306 cells/mm^3^; p < 0.0001), lower HIV-RNA VL (median 1.59 vs. 4.05 log_10_ copies/ml; p < 0.0001), and had been enrolled in EuroSIDA for a longer period (140.8 vs. 21.4 months; p < 0.0001). A higher proportion of participants who received RPV resided in Southern and West Central Europe (32.8% vs. 21.0% and 29.5% vs. 17.4%, respectively), with a similar proportion residing in the North (19.9% RPV, 20.4% EFV), while a far lower proportion of participants resided in Eastern Europe (2.1% vs. 23.1%; region global p = 0.0001). Other baseline characteristics and more detailed information are shown in Additional file 1: Table AF3. These differences detected by univariable analysis were confirmed in a multivariable logistic regression analysis. Adjusted Odds Ratio [aOR] for starting RPV-based vs. EFV-based regimens are shown in Table 2. Regarding the geographical distribution, after controlling for the other variables included in the model, participants treated with RPV were confimerd to be less likely to reside in East Europe (adjusted Odds Ratio [aOR]: 0.11 (95% confidence interval [CI]. 0.04–0.28; p < 0.001)) compared with West Central Europe.

**Table 1:** Demographic characteristics, comorbidities and medical conditions of participants initiating RPV or EFV-containing regimens

Characteristic	RPV N = 1355	EFV N = 333	p-value^1^
*Age*			
Median (IQR)	48.0 (40.0–54.0)	39.0 (32.5–47.0)	< 0.0001
*Weight (kg)*^2^			
Median (IQR)	73.8 (65.0–82.4)	70.2 (64.0–80.0)	0.1628
*Gender*			
Female	294 (21.7%)	53 (15.9%)	0.0193
Male	1061 (78.3%)	280 (84.1%)	
*Ethnicity*			
Non-white	214 (15.8%)	52 (15.6%)	0.9364
White	1141 (84.2%)	281 (84.4%)	
*Geographical Distribution*^*4*^			0.0001
South	445 (32.8%)	70 (21.0%)	
West Central	399 (29.4%)	58 (17.4%)	
North	269 (19.9%)	68 (20.4%)	
East Central	214 (15.8%)	60 (18.0%)	
East	28 (2.1%)	77 (23.1%)	
Mode of HIV transmission			0.0001
MSM	628 (46.3%)	116 (34.8%)	
PWID	318 (23.5%)	114 (34.2%)	
Heterosexual	316 (23.3%)	81 (24.3%)	
Other	93 (6.9%)	22 (6.6%)	
*Prior AIDS diagnoses*			
No	1036 (76.5%)	266 (79.9%)	0.1828
Yes	319 (23.5%)	67 (20.1%)	
*Prior non-AIDS diagnoses*			
Cardiovascular disease	59 (4.4%)	4 (1.2%)	0.0065
Non-AIDS defining malignancies	60 (4.4%)	6 (1.8%)	0.0267
End stage renal disease	5 (0.4%)	0 (0.0%)	0.2669
Pancreatitis	3 (0.2%)	0 (0.0%)	0.3901
*HCV antibody status*			
Negative	736 (54.3%)	127 (38.1%)	< 0.0001
Positive	541 (39.9%)	161 (48.3%)	
Unknown	78 (5.8%)	45 (13.5%)	
*HBsAg status*			
Negative	1133 (83.6%)	235 (70.6%)	< 0.0001
Positive	58 (4.3%)	19 (5.7%)	
Unknown	164 (12.1%)	79 (23.7%)	
*Diabetes*			
No	638 (47.1%)	124 (37.2%)	< 0.0001
Yes	91 (6.7%)	6 (1.8%)	
Unknown	626 (46.2%)	203 (61.0%)	
*Hypertension*			
No	768 (56.7%)	113 (33.9%)	< .0001
Yes	348 (25.7%)	48 (14.4%)	
Unknown	239 (17.6%)	172 (51.7%)	
*eGFR (mL/1.73m*^*2*^*)*			
Median (IQR)	99.2 (84.2–108.3)	106.9 (96.0–115.1)	< 0.0001
*CD4 cell count (/mm*^*3*^*)*			
Median (IQR)	566.0 (388.0–770.0)	305.5 (151.5–486.0)	< 0.0001
*CD4 cell count nadir (/mm*^*3*^*)*			
Median (IQR)	169.0 (35.0–283.0)	224.0 (76.0–330.0)	< 0.0001
*HIV viral load (log*_*10*_* copies/ml)*			
Median (IQR)	1.59 (1.28–1.69)	4.05 (1.60–5.11)	< 0.0001
*Time enrolled in EuroSIDA (months)*			
Median (IQR)	140.8 (69.4–209.7)	21.4 (1.3–89.2)	< 0.0001

IQR: Interquartile Range; BMI: Body Mass Index; MSM: Men who have sex with men; PWID: Injecting Drug User; HBsAg: Hepatitis B surface antigen; eGFR: estimated glomerular filtration rate; N/A: Not enough data to compute

^1^P-value for comparison of population distributions using the Kruskal–Wallis test or comparison of proportions using the chi-square test

^2^Baseline weight and eGFR are the closest measurements up to 1 year prior to baseline

^3^Participants can be included in EuroSIDA from the age of 16 years

^4^See Additional file 1: Fig. S5 for map of countries inclused in each geographical region

**Table 2:** Odds ratios of starting a RPV- vs. EFV-based regimen from fitting a logistic regression model

Factor	Logistic regression estimates of factors associated with initiating RPV vs EFV					
	Based on 1355 initiations of RPV and 333 initiations of EFV					
		Univariable estimates		Multivariable estimates		Type III p-value
		Odds ratio (95% CI)	p-value	Adjusted Odds ratio (95% CI)	p-value	
*Age/Sex/Race/Weigh*						
Age	per 5 years older	1.42 (1.33, 1.52)	< 0.001			
Male	vs. Female	0.68 (0.50, 0.94)	0.020	0.63 (0.34, 1.16)	0.137	
White	vs. Non-white	0.99 (0.71, 1.37)	0.936	1.68 (0.87, 3.22)	0.119	
Underweight (BMI < 19)	vs. (19 < = BMI < 25)	0.85 (0.48, 1.51)	0.588			
Overweight (25 < = BMI < 30)	vs. (19 < = BMI < 25)	1.36 (0.82, 2.26)	0.240			
Obese (BMI > = 30)	vs. (19 < = BMI < 25)	1.27 (0.55, 2.92)	0.572			
BMI unknown	vs. (19 < = BMI < 25)	0.46 (0.34, 0.63)	< 0.001			
*Geographical region*						< 0.001
South	vs. West Central	0.92 (0.64, 1.34)	0.679	0.64 (0.33, 1.24)	0.189	
North	vs. West Central	0.58 (0.39, 0.84)	0.005	0.45 (0.23, 0.91)	0.025	
East Central	vs. West Central	0.52 (0.35, 0.77)	0.001	0.43 (0.21, 0.91)	0.026	
East	vs. West Central	0.05 (0.03, 0.09)	< 0.001	0.11 (0.04, 0.28)	< 0.001	
*HIV Parameters*						
CD4 Cell count	per doubling	1.19 (1.13, 1.25)	< 0.001	1.37 (1.15, 1.63)	< 0.001	
CD4 Cell count nadir	per doubling	0.96 (0.91, 1.01)	0.119	0.79 (0.67, 0.92)	0.003	
HIV viral load	per log10 higher	0.46 (0.42, 0.51)	< 0.001	0.72 (0.61, 0.84)	< 0.001	
*HIV Transmission Route*						< 0.001
PWID	vs. MSM	0.52 (0.38, 0.69)	< 0.001	0.79 (0.43, 1.46)	0.458	
Heterosexuals	vs. MSM	0.72 (0.53, 0.99)	0.041	0.46 (0.26, 0.81)	0.007	
Other	vs. MSM	0.78 (0.47, 1.29)	0.337	0.56 (0.24, 1.29)	0.173	
*Hepatitis virus coinfection*						< 0.001
HBsAg positive	vs. Negative	0.63 (0.37, 1.08)	0.095	0.84 (0.27, 2.59)	0.766	
HBsAg unknown	vs. Negative	0.43 (0.32, 0.58)	< 0.001	1.49 (0.79, 2.78)	0.214	
HCVAb positive	vs. Negative	0.58 (0.45, 0.75)	< 0.001			
HCVAb unknown	vs. Negative	0.30 (0.20, 0.45)	< 0.001			
*Hypertension/Diabetes/eGFR*						
Previous hypertension	vs. None	1.07 (0.74, 1.53)	0.726	1.19 (0.70, 2.03)	0.519	< 0.001
Unknown hypertension	vs. None	0.20 (0.15, 0.27)	< 0.001	0.59 (0.31, 1.11)	0.100	
Previous diabetes	vs. None	2.95 (1.26, 6.88)	0.012	1.04 (0.33, 3.27)	0.942	< 0.001
Unknown diabetes	vs. None	0.60 (0.47, 0.77)	< 0.001	1.40 (0.88, 2.23)	0.155	
eGFR	per 5 mL/1.73m^2^	0.85 (0.81, 0.89)	< 0.001	0.94 (0.88, 1.00)	0.046	
*Prior AIDS diagnoses*						
Previous AIDS		1.22 (0.91, 1.64)	0.183	0.62 (0.34, 1.14)	0.125	
*Prior non-AIDS diagnoses*						
Cardiovascular disease		3.74 (1.35, 10.38)	0.011			
Non-AIDS defining malignancies		2.53 (1.08, 5.90)	0.032			
*Smoking Status*						< 0.001
Never smoked	vs. Curr smoker	1.26 (0.88, 1.81)	0.216	1.30 (0.78, 2.17)	0.322	
Former smoker	vs. Curr smoker	1.82 (1.02, 3.27)	0.044	0.87 (0.40, 1.93)	0.739	
Unknown smoking status	vs. Curr smoker	0.27 (0.20, 0.36)	< 0.001	1.24 (0.61, 2.50)	0.551	
*Time Controllers*						
Time Enrolled in EuroSIDA	per year longer	1.18 (1.15, 1.21)	< 0.001	1.17 (1.12, 1.23)	< 0.001	
*ART-status*						
ART-naïve	vs. ART-experienced	0.10 (0.08, 0.14)	< 0.001	0.86 (0.46–1.61)	0.634	

BMI Body mass index; MSM Men who have sex with men; PWID Injecting Drug User; HBsAgHepatitis B surface antigen; HCVAb Hepatitis C antibody; eGFR estimated glomerular filtration rate

Multivariable model includes all variables selected by backward selection that were retained with a p-value less than 0.3 level

### ARV treatment status

Overall, in the RPV group, 144 (10.6%) participants were ART-naive and 172 (12.7%) were naïve to cART (i.e. they had never previously received a regimen including ≥ 3 ARVs); overall 1,211 individuals (89.4%) had previously taken at least one ARV drug.

Additional file 1: Fig. AF1(a) shows the breakdown of the ARV treatment status of participants prior to initiating RPV- or EFV- containing regimens. A higher proportion of participants treated with RPV vs. EFV had been previously exposed to ARVs (i.e., exposure to at least 1 ARV from any class) (89.4% vs. 46.5%; p < 0.0001) and cART (i.e. exposure to ≥ 3 ARVs from any class) (87.3% vs. 42.6%; p < 0.0001) as compared to those who started EFV.

In the newly initiated RPV/EFV-based regimen,  the most frequently used NRTI pair was TDF/FTC (61%) followed by ABC/3TC (33%) and ZDV/3TC (2%) with no evidence for a difference in distribution by treatment group. Approximately 5% of the RPV group also received an INSTI (i.e. RAL or DTG) (Additional file 1: Fig. AF1(b)).

### Viral load and HIV drug resistance monitoring

Of the 1,355 participants who started an RPV-containing regimen, 1,184 (87.4%) had a VL measurement available in the 6 months prior to baseline. Of these, 938 (79.1%) had a VL ≤ 50 copies/ml, 235 (19.8%) had a VL between 51 and 100,000 copies/mL and 11 individuals (0.9%) had a VL > 100,000 copies/mL.

The median duration of virological follow-up for participants initiating RPV was 29.0 months (IQR: 15.6–41.6; range: 0–65.5) and 38.4 months (IQR: 17.2–52.7 range: 0–70.5) for those starting EFV. Median and IQR HIV-1 RNA at various follow-up times and up to 36 months from study entry are shown as box-plots stratified by treatment group in Additional file 1: Fig. AF2.

**Fig. 2:**
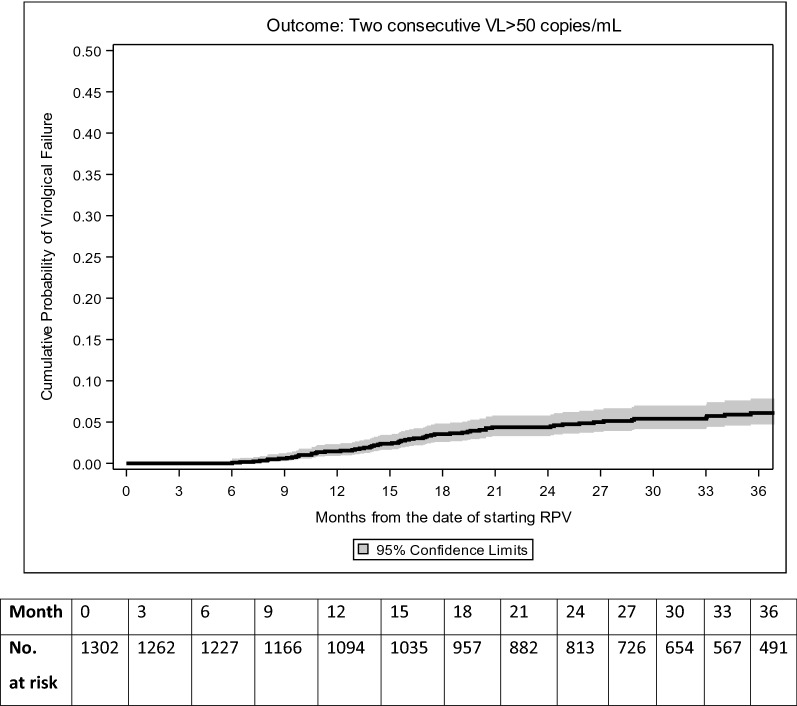
Kaplan Meier estimates of the risk of virological failure > 50 copies/mL in the RPV-recipient group

In the subset of 1,302/1,355 (96.1%) RPV and 323/333 (97.0%) EFV participants who had at least two VL values during follow-up, the proportion of those who experienced VF (two consecutive HIV-RNA > 50 copies/mL) was 5.5% (71/1,302, 95% CI 4.3–6.8%) and 7.4% (24/323, 95% CI 7.4–10.9%) for RPV- and EFV-based ART, respectively. The Kaplan–Meier estimates of the cumulative probability of VF for the RPV-based group were 1.5% (95% CI 0.7–2.2%) by 1 year and 4.5% (95% CI 3.3–5.7%) by 2 years from starting the RPV-based regimen (Fig. 2). Of note, this rate was mainly driven by the treatment-experienced group in whom the overall risk of VF was 66/1211 (5.5%). Due to the small number of events in the EFV group, the Kaplan–Meier estimates for participants who started the EFV-based regimens were not performed.

Pre-treatment screening for ARV RAMs was available for only 103/1,355 (8.3%) RPV-treated and 55/320 (17.2%) EFV-treated participants. Of the 103 RPV-treated participants with documented pre-treatment screening for ARV RAMs, 76 (73.8%) were treatment-experienced and 27 (26.2%) were treatment-naïve. The proportion of RPV- and EFV-treated participants with prior resistance testing who had at least 1 NNRTI RAM was 18/103 (17.5%) vs. 11/55 (20.0%), respectively. Among the 71 RPV-treated participants with VF, 15 (21.1%) had data available on treatment-emergent RAMs and 5 (33.3%) showed both NRTI and NNRTI RAMs, while 1 participant (6.7%) showed NNRTI RAMs but no NRTI RAMs. Thirteen out of 24 (54.2%) of the EFV-treated participants with VF had data available on treatment-emergent RAMs, and 3 of these (23.1%) showed both NRTI and NNRTI RAMs (Additional file 1: Tables AF4(a) an AF4(b) ). The breakdown of the individual treatment emergent NNRTI RAMs is also reported in these supplementary Tables. Unfortunately, none of the participants who experienced VF with resistance, had resistance test results available at baseline.

### Adverse events

In total, 498 (36.8%) of 1,355 participants initiating a RPV-containing regimen discontinued treatment during follow-up compared to 148 (44.4%) of 333 participants initiating a EFV-containing regimen.

Table 3 summarizes the reasons for discontinuing treatment. Discontinuations due to toxicity (combining all intolerance and toxicities reasons) were reported for 73 (14.7%) of RPV-treated participants compared with 45 (30.4%) of EFV-treated participants (p = 0.0001). Of note, toxicity of central nervous system (CNS) was significantly higher in participants who started EFV vs. RPV (22% vs. 3%, p < 0.001). Treatment failure (virological, immunological, and/or clinical) was the reason for discontinuation for 29 (5.8%) and 14 (9.5%) in the RPV and EFV groups, respectively (p = 0.03). In the RPV-treated group, 167 (33.5%) discontinued due to physician’s decision compared to 23 of 148 (15.5%) EFV-treated participants (p = 0.005). The proportion of discontinuations due to participants’ choice was 7.6% in the RPV-treated group compared to 12.2% in the EFV-treated group (p = 0.09). For 445/1,111 (40.1%) RPV-treated participants and 89/183 (48.6%) EFV-treated participants, ALT was reported to be above the normal range; and for 401/997 (40.2%) RPV-treated participants and 71/156 (45.5%) EFV-treated participants, AST was reported to be above the normal range (Table 4). There was no statistical evidence for a difference in AST between treatment groups (p>0.21).

**Table 3:** Reasons for discontinuing RPV or EFV

	RPV^1^ N (%)	EFV^2^ N (%)
Median (IQR) duration of treatment at discontinuation (Months)	18.2 (6.7–34.2)	9.8 (3.4–23.1)
Treatment failure (virological, immunological, and/or clinical)	29 (5.8)	14 (9.5)
Toxicity	73 (14.7)	45 (30.4)
Predominantly from central nervous system (CNS)	13 (2.6)	33 (22.3)
Predominantly from kidneys	20 (4.0)	4 (2.7)
Liver	6 (1.2)	3 (2.0)
Hypersensitivity reaction (skin eruption etc.)	3 (0.6)	2 (1.4)
Predominantly from abdomen/GI tract	13 (2.6)	1 (0.7)
Abnormal fat redistribution	2 (0.4)	0 (0.0)
Cardiovascular disease	2 (0.4)	0 (0.0)
Dyslipidaemia	0 (0.0)	1 (0.7)
Other side effects–not specified	14 (2.8)	1 (0.7)
Other		
Physician's decision, not specified	167 (33.5)	23 (15.5)
Participant's wish/decision, not specified	38 (7.6)	18 (12.2)
Availability of more effective treatment (not specifically failure or side effect related)	15 (3.0)	5 (3.4)
Structured Treatment Interruption (STI)	3 (0.6)	0 (0.0)
Enrolled in RCTs	2 (0.4)	0 (0.0)
Other causes, not specified	91 (18.3)	23 (15.5)
Unknown	80 (16.1)	20 (13.5)

Reasons listed are those for stopping either RPV or EFV

^1^Denominator is the number of participants who have discontinued RPV (n = 498)

^2^Denominator is the number of participants who have discontinued EFV (n = 148)

Reasons for discontinuation can be found at https://hicdep.org/Wiki/v/9/pt/4/Table/36/FieldID/439

**Table 4:** Frequency of laboratory abnormalities during the course of RPV or EFV treatment

Parameter Adverse event	RPV N (%)	EFV N (%)	p-value*
Haemoglobin^a^ (N with data: RPV = 691; EFV = 108)			
Below normal range	149 (21.6%)	41 (38.0%)	0.001
Normal range	488 (70.6%)	63 (58.3%)	
Above normal range	54 (7.8%)	4 (3.7%)	
ALT^b^ (N with data: RPV = 1111; EFV = 183)			
Normal range	666	94	
Above normal range	445 (40.1%)	89 (48.6%)	0.03
Above 3 times the normal range	85 (11.3%)	18 (16.1%)	0.15
AST^c^ (N with data: RPV = 997; EFV = 156)			
Normal range	596	85	
Above normal range	401 (40.2%)	71 (45.5%)	0.21
Above 3 times the normal range	69 (10.4%)	13 (13.3%)	0.39
ALP^d^ (N with data: RPV = 718; EFV = 127)			
Normal range	603	79	
Above normal range	115 (16.0%)	48 (37.8%)	< 0.0001
Above 3 times the normal range	2 (0.3%)	4 (4.8%)	0.003
Bilirubin^e^ (N with data: RPV = 1014; EFV = 156)			
Normal range	913	150	
Above normal range	101 (10.0%)	6 (3.9%)	0.01
Above 2 times the normal range	29 (3.1%)	3 (2.0%)	0.61
Platelets^f^ (N with data: RPV = 880; EFV = 127)			
Normal range	731	112	
Below normal range	149 (17.0%)	15 (11.8%)	0.16
Below 100 10^9^/L	58 (7.4%)	3 (2.6%)	0.07

^a^Haemoglobin normal range: (Men: 14.0 < g/dl < 18.0; Women: 12.0 < g/dl < 16.0)

^b^ALT normal range: (Men: U/L < 50; Women: U/L < 40)

^c^AST normal range: (Men: U/L < 40; Women: U/L < 34)

^d^ALP normal range: (Men: U/L < 128; Women: U/L < 98)

^e^Bilirubin normal range: (mg/dL < 1.4; μmol/L < 25.0)

^f^Platelets normal range: (140 < 10^9^/L < 400)

^&^Considering all values after baseline and while the person was still receiving the drug

^*^Chi-square p-value or Fisher exact test when < 5 events in the EFV group

^*^When two p-values are shown, they refer to separate 2 × 2 tables with the ‘Normal range’ category used as common comparator

## Discussion

We have assessed clinical characteristics, virological outcomes, emergence of RAMs and adverse events during follow up, for individuals who initiated a RPV-or EFV-containing regimen. Over the period November 2011-December 2017, the majority (99%) of individuals initiating a RPV-containing regimen, did so with a HIV-RNA ≤ 100,000 copies/ml, in accordance with the approved EU labelled indication. Overall, although a formal comparison with EFV was not perfomed  as by the pre-specified protocol, we found that rate of VF and development of RAMs by 2 years was low in the RPV group (23%) and rates were comparable to those experienced by PLWH who started EFV (33%). Of note, the frequency of discontinuation due to toxicity was lower in RPV vs. EFV (15% versus 30%).

There were some key differences in the case-mix of participants who started RPV-based regimens vs. EFV-based regimens in the study. First, there were significant regional differences with participants treated with RPV less likely to reside in East Europe compared with West Central Europe. Participants starting RPV-based regimens also typically had lower HIV-1 RNA levels and higher CD4 counts. Interestingly, there was no evidence for a difference in the prevalence of ART-naïve participants by treatment group after controlling for other key predictors.

Protocol targets for the number of VF required to perform a formal comparison could not be achieved because the number of participants who started EFV and recruited in the study decreased over time, most likely because the European treatment guidelines starting from 2015 ceased to recommend its use. However, some conclusions might be drawn by comparing the VF estimates in the RPV group with those of historical data [2–9, 14, 15]. In the majority of the previously conducted randomized comparisons, EFV was the control group and, in these settings, no difference in rate of VF was found between RPV and EFV overall or in subsets of individuals (e.g. people with HBV co-infection). Even when considering the upper limit of confidence interval of 6% VF by 2 years for RPV recipients, this estimated rate is lower than that observed in participants enrolled in randomized studies [2–9]. The slight discrepancy in the rate of RPV VF in our cohort vs. that observed in the ECHO/THRIVE trials (5.4% failure by 1 year) might be due to the fact that the trial participants used single compound combination therapy while in our analysis a large proportion of participants used the FDC Eviplera™. Indeed, Study GS-US-264-0106 (SPIRIT), conducted in participants with VL ≤ 50 copies/mL who were switched to RPV- based FDC, showed a more comparable rate of VF of 11% by 1 year [9]. Of note, most of  these clinical trials were conducted in ART-naïve participants, while our analysis uses the data of participants who started RPV when they were ART-experienced, comprising a selected population at reduced risk of VF. A summary of the rates of RPV VFs by 1 year in EuroSIDA as well as in the mentioned randomized clinical trials [2–9] is shown in a Additional file 1: Table AF5 which also includes other estimates from real-world studies in Europe [10–12].

Resistance test results were available for a small subset (21%) of RPV participants who experienced VF. Detection of genotypic resistance in this subset was infrequent and the profile of individual treatment-emergent NRTI and NNRTI RAMs among participants on RPV-based regimens was that expected in those failing these combinations. Participants in whom resistance mutations were detected at time of failure did not have a baseline resistance test available.  However, the prevalence of baseline NNRTI-associated resistance estimated from those with available data was lower than 20%; thus, we can speculate that for the majority of participants, the mutations detected at VF have been newly acquired rather than pre-exist, which is consistent with the results of previously published reports [2–9]. Nevertheless, in Study GS-US-264-0110 (STaR), 6% of participants starting RPV in FDC developed HIV-drug resistance by 96 weeks of follow-up, which was mainly seen within the first 48 weeks [5–7]; of note, this rate was significantly higher than that seen for EFV (2.3%), but our data did not confirm this finding.

Overall 37% of participants initiating RPV discontinued the drug compared to 44% in those initiating EFV. RPV led to less discontinuation due to toxicity (14.7% vs. 30.4%), especially for CNS toxicity, which is a recognized side effect for EFV [18]. There was a higher probability of stopping RPV due to physician’s choice (33.5% vs. 15.5%), possibly indicating modifications due to proactive switches, risk of drug to drug interactions, problems associated with food intake, planning of pregnancy or possibly reported poor adherence. Although misclassification of the reason for stopping is always possible, treatment toxicity and VF are specifically listed as one of the reasons in EuroSIDA RedCap, so it is unlikely that those were recorded as ‘physician’s choice’. Moreover, only adverse events leading to drug discontinuations are recorded in EuroSIDA and a number of low grade adverse events are not routinely recorded. Of note, because EFV has a more recognized toxicity profile, it is possible that for a given symptom severity EFV had a greater risk of being discontinued than RPV. However, it could be equally argued that because RPV was a newer drug, monitoring of toxicity might have been stricter for participants receiving RPV. The frequency of discontnuations due to VF were higher in the RPV group but this was not analysed by means of the more reliable analysis looking at the actual viral load values. Drug formulation are known to also have an effect on adherence and hence effectiveness/outcomes. Nevertheless, cases in which drugs were used as FDC vs. combinations of single compounds have been treated equally in this analysis. In addition, no difference in laboratory abnormalities between RPV and EFV when comparing the proportion of participants with a value below/above normal values for ALT and AST were found. These data confirm the safety profile of RPV observed in the trials [2–9].

This study has several limitations. First, since allocation of treatment in observational studies is not randomised, treatment decisions may be influenced by prognostic factors and the resulting imbalance in the underlying risk profile between groups can generate biased results. Some of these unmeasured confounders may include adherence to treatment, minor symptoms and socio-demographic factors. Importantly, most comparisons were conducted in unadjusted analysis or, like for the virological endpoint, using historical data so measured confounding cannot be excluded either. The slow recruitment of new participants starting EFV could not be predicted and ultimately limited the ability to perform a formal comparison between the two groups. Moreover, resistance data collection stopped in EuroSIDA in March 2017 so cumulative estimates of resistance detection are likely to be underestimated. Finally, the analysis includes EuroSIDA data collected up to 2018 and this is the reason why more contemporary RPV regimens such as those based on TAF have not been included.

Key strengths of the study are the real-world setting, its large sample size (larger than any other cohort study published to date for the ART-experienced population treated with RPV-based regimens) and the average two years of virological follow-up. Another strength is the prospective nature of the EuroSIDA cohort so that reverse causality is unlikely to have occurred for the main endpoint of VF/development of resistance as this temporarily occured after the initiation of RPV or EFV. Of note, the results and conclusions from this analysis are not directly generalizable outside of the subset of EuroSIDA  participants satisfying the inclusion criteria for this analysis.

In conclusion, RPV was used in accordance with the approved indication for RPV-containing regimen in Europe. Even in the worst-case scenario (upper limit of confidence interval) our real-world estimate of the rate of VF > 50 copies/mL in participants treated with RPV were similar to those reported by other European cohorts and even lower than the rates observed in randomized clinical studies. Based on available data, the proportion of participants who acquired NNRTI resistance-associated mutations was low in the few participants who experienced VF in both treatment groups. Importantly, this data also confirm the safety profile of RPV, with less frequent discontinuation due to toxicity than EFV (especially for CNS toxicity) and no evidence for a difference in liver toxicity, consistent with what was observed in randomized trials. The potential low cost and dose of the drug means that rilpivirine can potentially be manufactured at a low price. Moreover, its long half-life suggests the potential for monthly dosing via nonoral routes, with promising early results from studies of a long-acting injectable formulation. These characteristics still currently make rilpivirine an attractive drug for resource-limited settings.

## Supplementary Information

**Additional file 1.** Key defintions and additional results mentioned in the text which are not included in the main Tables and Figures.

## Data Availability

Access to the EuroSIDA Data: The EuroSIDA Steering Committee encourages the submission of concepts for research proposals. Concepts can be submitted for review using an online research concept, please see our website (https://www.chip.dk). The concept is evaluated by the Steering Committee for scientific relevance, relevance to the EuroSIDA study, design, feasibility and overlap with already approved projects. All proposers will receive feedback and revision of the concept may be requested. If approved, a writing group will be established consisting of proposers, members of the Steering Committee and staff at the coordinating center and the statistical center.
