# Supplementary material for: Observational cohort study of rilpivirine (RPV) utilization in Europe
Source: AIDS Res Ther. 2022 Aug 6;19:38. doi: 10.1186/s12981-022-00457-0 (PMC9357334; doi:10.1186/s12981-022-00457-0)
Supplement: Supplementary file 1 — Additional file 1. Key defintions and additional results mentioned in the text which are not included in the main Tables and Figures. [file 12981_2022_457_MOESM1_ESM.docx]

**Additional file**

Key Definitions

Geographical region: 1) South = Greece, Italy, Portugal, Spain; 2) West Central = Belgium, France, Germany, Luxembourg, Switzerland, Austria; 3) North = Denmark, UK, Finland, Ireland, The Netherlands, Sweden, Norway, Iceland; 4) East Central = Hungary, Czech Republic, Slovakia, Bulgaria, Poland, Romania, Croatia; 5) East = Estonia, Latvia, Lithuania;

NNRTI-associated resistance mutations: V090I, A098G, L100I, K101E, K101H, K101P, K103N, K103S, V106A, V106I, V106M, V108I, E138A, E138G, E138K, E138Q, E138R, V179D, V179F, V179L, V179T, Y181C, Y181I, Y181V, Y188C, Y188H, Y188L, G190A, G190S, H221Y, P225H, F227C, M230L

NRTI-associated resistance mutations: M41L, A62V, K65R, D67N, K70E, K70R, L74V, V75I, F77L, Y115F, F116Y, Q151M, M184I, M184V, L210W, T215F, T215Y, K219E, K219Q

RPV resistance-associated mutations: K101E, K101P, E138A, E138G, E138K, E138Q, E138R, V179L, Y181C, Y181I, Y181V, Y188L, H221Y, F227C, M230I, M230L

EFV resistance-associated mutations: L100I, K101P, K103N, K103S, V106M, V108I, Y181C, Y181I, Y188L, G190S, G190A, P225H, M230L

List of EuroSIDA countries where RPV is registered per the indication in the EU Summary of Product Characteristics (i.e. participants with a baseline viral load of ≤100,000 copies/ml)

Austria Belgium Bulgaria

Czech Republic Denmark Estonia

Finland France Germany Greece Hungary Iceland Ireland Italy Latvia Lithuania

Luxembourg Netherlands Poland Portugal Romania Slovakia Spain Sweden

UK*

Norway Croatia

*UK was still included in the EU at the time of analysis

**Additional file 1: Table AF1: Proportion of participants treated with rilpivirine (RPV) in accordance with the summary of product characteristics**

|  | **Total treated with RPV (N=1355)** |
| --- | --- |
| Number of participants cART-naive^1^ | 172 (12.7%) |
| Number of treatment naive participants with HIV viral load ≤100,000 copies/ml^1,^ ^2^ | 140 (95.2%) |
|  |  |
| Number of participants with documented pre-treatment screening for ARV RAMS^3^ | 103 (7.6%) |
| Number of treatment naive participants with pre-treatment^&^ screening for ARV  RAMS^3,^ ^4^ | 27 (26.2%) |
| Number of treatment experienced participants with pre-treatment^&^ screening for ARV RAMS^3,^ ^4^ | 76 (73.8%) |
|  |  |
| Number of participants with baseline HIV viral load ≤100,000 copies/ml^5^ | 1173 (99.1%) |
| Number of participants with baseline HIV viral load ≤50 copies/ml^6^ | 938 (80.0%) |

RPV: Rilpivirine; ARV: Antiretroviral; RAM: Resistance Associated Mutation

Baseline HIV viral load defined as the nearest viral load measurement up to 6 months prior to baseline or 3 months after

^&^It refers to the 5 years before treatment initiation Baseline HIV RNA data are missing for 171 participants ^1^cART defined as ≥3 ARVs

^2^Denominator is the number of treatment naive participants with known baseline HIV viral load (n=147)

^3^Including resistance screening up to 5 years prior to baseline

^4^Denominator is the number of participants with pre-treatment screening for ARV RAMS (n=103) ^5^Denominator is the number of participants with known baseline HIV viral load (n=1184) ^6^Denominator is the number of participants with baseline HIV viral load ≤100,000 copies/ml (n=1173).

**Additional file 1: Table AF2: Demographic characteristics, comorbidities and medical conditions of participants initiating RPV or EFV-containing regimens**

| **Characteristic** | **RPV N=1355** | **EFV N=333** | **P-value^1^** |
| --- | --- | --- | --- |
| ***Age Categories*^3^** | | | |
| 16 ≤ Age < 18 | 1 (0.1%) | 1 (0.3%) | 0.0026 |
| 18 ≤ Age < 65 | 1274 (94.0%) | 327 (98.2%) |  |
| Age ≥ 65 | 80 (5.9%) | 5 (1.5%) |  |
|  | | | |
| ***Weight (kg)*^2^** | | | |
| N | 720 | 109 |  |
| Median (IQR) | 73.8 (65.0 - 82.4) | 70.2 (64.0 - 80.0) | 0.1628 |
|  | | | |
| ***BMI*** | | | |
| Underweight (BMI <19) | 95 (7.0%) | 18 (5.4%) | <.0001 |
| Normal Weight (19 ≤ BMI < 25) | 377 (27.8%) | 61 (18.3%) |  |
| Overweight (25 ≤ BMI < 30) | 193 (14.2%) | 23 (6.9%) |  |
| Obese (BMI ≥ 30) | 55 (4.1%) | 7 (2.1%) |  |
| BMI Unknown | 635 (46.9%) | 224 (67.3%) |  |
|  |  |  |  |
| ***Smoking status*** |  |  |  |
| Never | 472 (34.8%) | 63 (18.9%) | <.0001 |
| Current | 429 (31.7%) | 72 (21.6%) |  |
| Former | 163 (12.0%) | 15 (4.5%) |  |
| Unknown | 291 (21.5%) | 183 (55.0%) |  |
|  |  |  |  |
| ***eGFR*^2^** |  |  |  |
| eGFR < 60 | 54 (4.0%) | 2 (0.6%) | <.0001 |
| 60 ≤ eGFR < 90 | 305 (22.5%) | 25 (7.5%) |  |
| eGFR ≥ 90 | 764 (56.4%) | 181 (54.4%) |  |
| eGFR Unknown | 232 (17.1%) | 125 (37.5%) |  |

IQR: Interquartile Range; BMI: Body Mass Index;

eGFR: estimated glomerular filtration rate;

^1^P-value for comparison of population distributions using the Kruskal-Wallis test or comparison of proportions using the chi-square test

^2^Baseline weight and eGFR are the closest measurements to baseline up to 1 year prior

^3^Participants can be included in EuroSIDA from the age of 16 years

**Additional file 1: Table AF3(a):**

**Treatment emergent NNRTI resistance associated mutations in participants initiating RPV or EFV**

***N participants tested for***

***treatment emergent RAMs***

| ***Reverse transcriptase***  ***Amino Acid Position*** | ***RPV*** | ***EFV*** |
| --- | --- | --- |
| ***Mutation*** | ***(N=15)*** | ***(N=13)*** |
| **N(%) participants with at least 1 NNRTI RAM** | 6 (40.0%) | 3 (23.1%) |
| **N(%) participants with at least 1 RPV RAM** | 2 (13.3%) | 0 (0.0%) |
| **N(%) participants with at least 1 EFV RAM** | 3 (20.0%) | 3 (23.1%) |
| **Position 90** |  |  |
| V90I | 2 (13.3%) | 0 (0.0%) |
| **Position 98** |  |  |
| A98G | 0 (0.0%) | 1 (7.7%) |
| **Position 100** |  |  |
| L100I** | 0 (0.0%) | 0 (0.0%) |
| **Position 101** |  |  |
| K101E* | 0 (0.0%) | 0 (0.0%) |
| K101H | 0 (0.0%) | 0 (0.0%) |
| K101P*/** | 0 (0.0%) | 0 (0.0%) |
| **Position 103** |  |  |
| K103N** | 3 (20.0%) | 3 (23.1%) |
| K103S** | 0 (0.0%) | 0 (0.0%) |
| **Position 106**  V106A | 1 (6.7%) | 0 (0.0%) |
| V106I | 0 (0.0%) | 0 (0.0%) |
| V106M** | 0 (0.0%) | 0 (0.0%) |
| **Position 108** |  |  |
| V108I** | 0 (0.0%) | 0 (0.0%) |
| **Position 138** |  |  |
| E138A* | 0 (0.0%) | 0 (0.0%) |
| E138G* | 0 (0.0%) | 0 (0.0%) |
| E138K* | 1 (6.7%) | 0 (0.0%) |
| E138Q* | 0 (0.0%) | 0 (0.0%) |
| E138R* | 0 (0.0%) | 0 (0.0%) |
| **Position 179** |  |  |
| V179D | 0 (0.0%) | 0 (0.0%) |

**Additional file 1: Table AF3(a)(cont.)**

**Treatment emergent NNRTI resistance associated mutations in participants initiating RPV or EFV**

***N participants tested for***

***treatment emergent RAMs***

| ***Amino Acid Position*** | ***RPV*** | ***EFV*** |  |
| --- | --- | --- | --- |
| ***Mutation*** | ***(N=15)*** | ***(N=13)*** |  |
| V179F | 0 (0.0%) | 0 (0.0%) |  |
| V179L* | 1 (6.7%) | 0 (0.0%) |  |
| V179T | 1 (6.7%) | 0 (0.0%) |  |
| **Position 181**  Y181C*/** | 0 (0.0%) | 0 (0.0%) |  |
| Y181I*/** | 0 (0.0%) | 0 (0.0%) |  |
| Y181V* | 0 (0.0%) | 0 (0.0%) |  |
| **Position 188** |  |  |  |
| Y188C | 0 (0.0%) | 0 (0.0%) |  |
| Y188H | 0 (0.0%) | 0 (0.0%) |  |
| Y188L*/** | 0 (0.0%) | 0 (0.0%) |  |
| **Position 190** |  |  |  |
| G190A** | 0 (0.0%) | 0 (0.0%) |  |
| G190S** | 0 (0.0%) | 0 (0.0%) |  |
| **Position 221** |  |  |  |
| H221Y* | 0 (0.0%) | 0 (0.0%) |  |
| **Position 225**  P225H** | 0 (0.0%) | 3 (23.1%) |  |
| **Position 227** |  |  |  |
| F227C* | 0 (0.0%) | 0 (0.0%) |  |
| **Position 230** |  |  |  |
| M230I* | 1 (6.7%) | 0 (0.0%) | |
| M230L* | 1 (6.7%) | 0 (0.0%) | |

RAM: Resistance associated mutation.

^1^RPV RAMs are indicated with an asterisk (*); EFV RAMs are indicated with a double asterisk (**) There were 0 participants who have both the L100I and K103N mutations detected at time of virological failure

**Additional file 1: Table AF3(b):**

**Treatment emergent NRTI resistance associated mutations participants initiating RPV or EFV**

***N participants tested for***

***treatment emergent RAMs***

| ***Reverse Transcriptase***  ***Amino Acid Position*** | ***RPV*** | ***EFV*** |
| --- | --- | --- |
| ***Mutation*** | ***(N=15)*** | ***(N=13)*** |
| **N(%) participants with at least 1 NRTI RAM** | 5 (33.3%) | 3 (23.1%) |
| **Position 41** |  |  |
| M41L | 1 (6.7%) | 0 (0.0%) |
| **Position 62** |  |  |
| A62V | 1 (6.7%) | 0 (0.0%) |
| **Position 65**  K65R | 1 (6.7%) | 0 (0.0%) |
| **Position 67** |  |  |
| D67N | 1 (6.7%) | 1 (7.7%) |
| **Position 70**  K70E | 1 (6.7%) | 0 (0.0%) |

K70R 1 (6.7%) 0 (0.0%)

**Position 74**

L74V 0 (0.0%) 0 (0.0%)

**Position 75**

V75I 0 (0.0%) 0 (0.0%)

**Position 77**

F77L 0 (0.0%) 0 (0.0%)

**Position 115**

Y115F 0 (0.0%) 0 (0.0%)

**Position 116**

F116Y 0 (0.0%) 0 (0.0%)

**Position 151**

Q151M 0 (0.0%) 0 (0.0%)

| **Position 184** |  | |
| --- | --- | --- |
| M184I | 2 (13.3%) | 0 (0.0%) |
| M184V | 1 (6.7%) | 3 (23.1%) |
| **Position 210** |  |  |
| L210W | 0 (0.0%) | 0 (0.0%) |
| **Position 215** |  |  |
| T215F | 0 (0.0%) | 0 (0.0%) |
| T215Y | 1 (6.7%) | 0 (0.0%) |
| **Position 219** |  |  |
| K219E | 0 (0.0%) | 0 (0.0%) |
| K219Q | 1 (6.7%) | 0 (0.0%) |

RAM: Resistance associated mutation. All mutations are within the reverse transcriptase

**Additional file 1: Table AF4:**

**Rates of RPV virological failure from published randomized clinical trials and observational studies**

| **Study** | **Nature** | **Population** | **Baseline VL**  **(copies/mL)** | **Sample size** | **Rate of RPV VF at**  **1 year** | **95% CI** |
| --- | --- | --- | --- | --- | --- | --- |
| EuroSIDA | Real-  life | ART-naïve and  ART-experienced | ≤100,000 | 1302 | 1.5% | 0.7-2.2% |
| Study C209 (ECHO) and Study C215 (THRIVE),  pooled [[3](#_bookmark104),[4](#_bookmark105)] | RCT | ART-naive | >5,000 | 686 | 14% | 11.5-16.8% |
| Study C209 (ECHO) and Study C215 (THRIVE),  subset with baseline VL  ≤100,000 copies/ml [[4](#_bookmark104)] | RCT | ART-naive | ≤100,000 | 368 | 5.4% | 3.4-8.3% |
| Study GS-US-264-0111  [[5](#_bookmark106)] | RCT | ART-experienced | ≤50 | 49 | 4.1% | 0.5-14.0% |
| Study GS-US-264-0110  (StaR) [[6](#_bookmark107),7] | RCT | ART-naïve (FDC  combinations) | <50 | 394 | 6.1% | 3.9-8.9% |
| Study GS-US-264-0106 (SPIRIT) [[8](#_bookmark109)] | RCT | ART-experienced (FDC  combinations) | <50 | 476 | 10.7% | 7.5-14.7% |
| Icona [1[5](#_bookmark110)] | Real-  life | ART-naive |  | 786 | 1.8% | 1.0-2.3% |
| Italian 5-center study  [1[6](#_bookmark111)] | Real-  life | ART-experienced |  | 463 | 7.3* | 4.8-9.8%* |
| Swiss Cohort [1[7](#_bookmark112)] | Real-  life | ART-naïve and  ART-experienced |  | 644 | 2.2% | 1.1-3.7% |

**3-year estimates*

**Additional file 1: Table AF5. Codebook of variables included in the logistic regression model**

| **Variable** | **Variable name** | **Type** | **Model fit** | **Interpretation of OR** |
| --- | --- | --- | --- | --- |
| Age | ‘Age5’ | Continuous | Value divided by 5 | OR change for 5 years older age |
| Gender | ‘Male’ | Binary | Male vs. Female | OR comparing male vs female |
| Ethnicity | ‘White’ | Binary | White vs non-white | OR comparing white vs. non-white |
| BMI | ‘Under’  ‘Over’ ‘Obese’ ‘Unk’ | Categorical | 5 groups:  <19  19-25 (reference)  25-30  >30  Unknown | OR comparing all groups with the 19-25 group |
| Geographical region  (see Figure below) | ‘South’  ‘Eastc’ ‘East’  ‘North’ | Categorical | 5 groups:  South  Western (ref)  East central  East  North | OR comparing all groups with Western |
| CD4 count | ‘L2_cd4’ | Continuous | log2 scale | OR change for a doubling of CD4 count |
| Nadir CD4 count | ‘L2_cd4n’ | Continuous (minimum value ever recorded prior to baseline) | log2 scale | OR change for a doubling of nadir CD4 count |
| HIV-RNA | ‘Base_rna’ | Continuous | log10 | OR change for a log10 higher HIV-RNA |
| Mode of HIV transmission | ‘Idu’  ‘Hetero’  ‘other’ | Categorical | 4 groups:  MSM (reference)  PWID  Heterosexual  Other | OR comparing all groups with MSM |
| Hepatitis B co-infection | ‘Hbv_pos’  ‘Hbv_unk’ | Categorical | 3 serology groups:  HBsAg-negative (ref)  HBsAg-positive  Unknown | OR comparing all groups with HBsAg-negative |
| Hepatitis C co-infection | ‘Hcv_pos’  ‘Hcv_unk’ | Categorical | 3 serology groups:  HCVAb -negative (ref)  HCVAb -positive  Unknown | OR comparing all groups with HCVAb -negative |
| Hypertension | ‘Hyp_pos’  ‘Hyp_unk’ | Categorical | 3 groups:  No hypertension (ref)  Previous hypertension  Unknown | OR comparing all groups with no-hypertension |
| Diabetes | ‘Diab_pos’  ‘Diab_unk’ | Categorical | 3 groups:  No diabetes (ref)  Previous diabetes  Unknown | OR comparing all groups with diabetes |
| eGFR | ‘egfr5’ | Continuous  (CKD-EPI equation) | Value divided by 5 | OR change for 5 points higher EGFR |
| Previous AIDS | ‘p_AIDS’ | Binary | Yes vs. No | OR for AIDS vs. not AIDS |
| Cardiovascular Disease | ‘CVD’ | Binary | Yes vs. No | OR for CVD vs. not CVD |
| Non-AIDS malignancies | ‘p_nadm | Binary | Yes vs. No | OR for malignancies vs. not |
| Smoking | ‘never’  ‘former’  ‘unk_s’ | Categorical | 4 groups:  Never  Former  Current (ref)  Unknown | OR comparing all groups with current smoker |
| Time from entry in EuroSIDA | ‘time_euro’ | Continuous | Untransformed value | OR per 1 year longer in the study |
| ART status | ‘art_naive’ | Binary | Naïve vs Experienced | OR for ART-naïve vs. experienced |

|  |
| --- |

**Additional file 1: Figure. AF1.** Antiretroviral use by treatment group (percentage)

1. Before baseline
2. Breakdown of NRTI-pairs and other anchor drug used with RPV or EFV

**Additional file 1: Figure. AF2:**

**Median (IQR) HIV Viral Load Through Time in Participants**


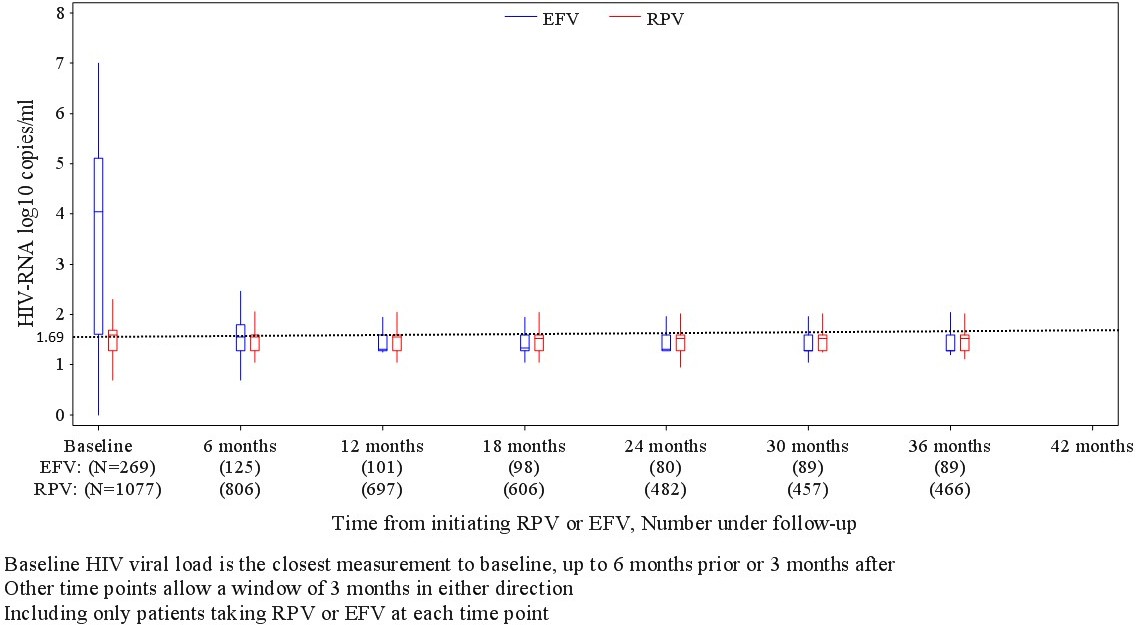
**Initiating RPV or EFV**

NB Horizontal dotted line indicated the threshold of 50 copies/mL in the log_10_ scale

(1.69 log_10_ copies/ml)

**Additional file 1: Figure. AF3. Map of current EuroSIDA clinics, countries and regions.**


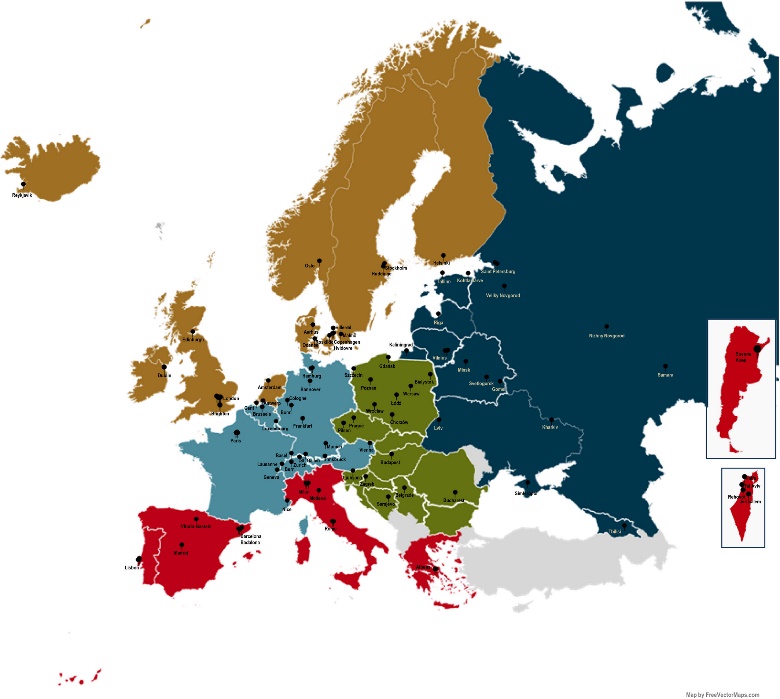


Countries are grouped into the following regions: *Western Europe (light blue)*: Austria, Belgium, France, Germany, Luxembourg and Switzerland. *Southern Europe (red)*: Argentina, Greece, Israel, Italy, Portugal and Spain. *Northern Europe (brown)*: Denmark, Finland, Iceland, Ireland, the Netherlands, Norway, Sweden and the UK. *East Central Europe (green)*: Bosnia-Herzegovina, Croatia, the Czech Republic, Hungary, Poland, Romania, Serbia and Slovenia. *Eastern Europe (dark blue)*: Belarus, Estonia, Georgia, Latvia, Lithuania, Russia and Ukraine. An up-to-date list of the EuroSIDA study group may be found on the website: <http://www.chip.dk/Studies/EuroSIDA/Study-group>.
